# Sympatric ecological divergence associated with a color polymorphism

**DOI:** 10.1186/s12915-015-0192-7

**Published:** 2015-10-05

**Authors:** Henrik Kusche, Kathryn R. Elmer, Axel Meyer

**Affiliations:** Department of Biology, University of Konstanz, 78457 Konstanz, Germany; International Max Planck Research School for Organismal Biology, University of Konstanz, 78457 Konstanz, Germany; Present address: Département de Biologie, Institut de Biologie Intégrative et des Systèmes (IBIS) Université Laval, 1030 Avenue de la Médecine, Québec, G1V 0A6 QC Canada; Present address: Institute of Biodiversity, Animal Health & Comparative Medicine, College of Medical, Veterinary & Life Sciences, University of Glasgow, Glasgow, G12 8QQ UK

**Keywords:** Adaptive radiation, Ecological diversification, Evolutionary ecology, Parallel evolution, Color polymorphism, Stable isotope analysis, Crater lake, Cichlids, Genetic correlation, Pleiotropy, Genetic linkage

## Abstract

**Background:**

Color polymorphisms are a conspicuous feature of many species and a way to address broad ecological and evolutionary questions. Three potential major evolutionary fates of color polymorphisms are conceivable over time: maintenance, loss, or speciation. However, the understanding of color polymorphisms and their evolutionary implications is frequently impaired by sex-linkage of coloration, unknown inheritance patterns, difficulties in phenotypic characterization, and a lack of evolutionary replicates. Hence, the role of color polymorphisms in promoting ecological and evolutionary diversification remains poorly understood. In this context, we assessed the ecological and evolutionary consequences of a color polymorphic study system that is not hampered by these restrictions: the repeated adaptive radiations of the gold/dark Midas cichlid fishes (the *Amphilophus citrinellus* species complex) from the great lakes and crater lakes of Nicaragua, Central America.

**Results:**

We conducted multi-trait morphological and ecological analyses from ten populations of this young adaptive radiation (<6,000 years old), which revealed sympatric ecological differentiation associated with the conspicuous binary (gold/dark) color polymorphism. Varying degrees of intraspecific ecological divergence were observed across the ten color morph pairs, but most pairs exhibited a consistently parallel ecological and evolutionary trajectory across populations. Specifically, gold Midas cichlids are frequently deeper-bodied, have more robust pharyngeal jaws, and feed at a lower trophic level compared to conspecific, sympatric dark individuals. A common garden experiment suggests there is a genetic correlation of color and eco-morphological traits.

**Conclusions:**

We demonstrate unprecedented ecological and evolutionary consequences of color polymorphism in this adaptive radiation. Across the species complex, sympatric conspecific individuals differed in eco-morphology depending on color morph (gold/dark) and the axis of differentiation tended to be consistent across replicates. The consistent divergence across wild populations and the common garden experiment suggests that color is genetically correlated to ecology. Because Midas cichlids are known to mate color assortatively, the putative genetic correlation of this color polymorphism with an eco-morphological divergence suggests an innate potential to promote ecological and evolutionary divergence across this species complex. However, there are to date no examples of speciation based on color in this radiation, suggesting long-term maintenance of this color polymorphism.

**Electronic supplementary material:**

The online version of this article (doi:10.1186/s12915-015-0192-7) contains supplementary material, which is available to authorized users.

## Background

Color polymorphisms, or the occurrence of two or more genetically determined color morphs within an interbreeding population [1], occur in animals and plants alike. They potentially have a great impact on various intra- and interspecific processes such as communication, mating systems, and vulnerability to predation [2–4], and, consequently, may accelerate speciation [5]. Ultimately, color polymorphisms can be maintained in the population, they can disappear from the population, and under particular circumstances may facilitate sympatric diversification.

Given the prevalence of color polymorphisms throughout the tree of life, it is surprising that their significance in promoting ecological and evolutionary diversification has been rarely demonstrated empirically [3, 5, 6]. The understanding of color polymorphism still remains incomplete because of the complexities associated with its development in many systems. For example in complex animal systems, color polymorphisms tend to have unknown inheritance mechanisms, exhibit continuous or intermediate variation, result from assortative mating under natural conditions, and/or be sex-linked [3] as seen in the African cichlid fishes [7].

Theory predicts that sympatric divergence can occur rapidly when ecological traits under divergent natural selection are genetically correlated and affect mate choice [3, 8–10], hereinafter referred to as “genetic correlation”. Genetic correlation does not imply that the genetic architecture of the trait is fully resolved. It may include single-locus “magic traits” but also cases where there is tight physical genetic linkage of the mate choice genes and the genes that affect ecology, which effectively imitate a true “magic trait” [8]*.* However, empirical evidence for genetic correlation is exceedingly rare. Some classic examples in animals include assortative mating by body size in stickleback species pairs [11] and by body shape in *Gambusia* mosquitofish [12]. Even fewer studies suggest such genetic correlations between mate choice relevant traits and those under divergent selection in color polymorphic systems, as has been found in African cichlid fish [13].

**Fig. 1:**
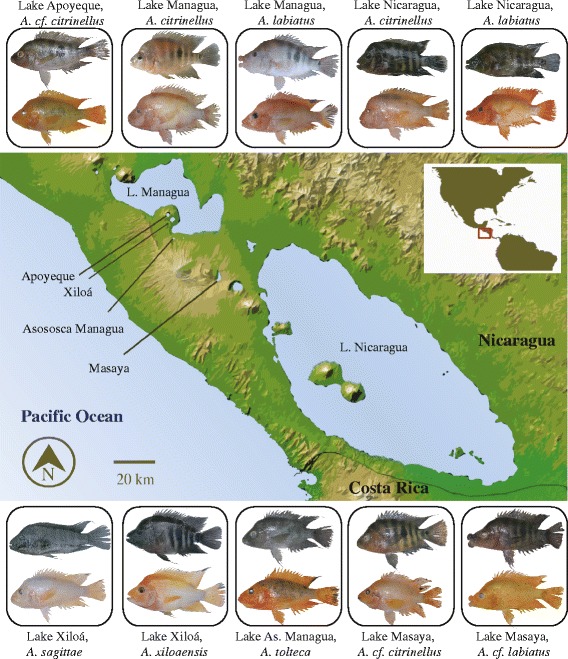
Geographic distribution of color morph pairs in the young adaptive radiation of Midas cichlids. In Nicaragua, a number of small crater lakes have been colonized by Midas cichlids from the great lakes Managua and Nicaragua. The Midas cichlids within each crater lake are genetically more similar to each other than to any other population [14]. Gold morphs are found in both great lake and most crater lake populations, usually at low frequencies [17, 19]

An ideal study system to address the ecological and evolutionary consequences of color polymorphisms would incorporate multiple replicates, sex-independence, and a simple genetic basis of the color polymorphism associated with assortative mating. For the first time, we assessed the ecological divergence in a color polymorphic system that meets all these advantageous criteria: the adaptive radiation of Midas cichlid fishes (the *Amphilophus citrinellus* species complex) from the great lakes and crater lakes of Nicaragua (Fig. 1). Midas cichlids have become a model system for studying parallel adaptive radiation and ecological speciation. In western Nicaragua, several crater lakes have been formed by accumulation of rain and ground water in isolated volcanic calderas. In rare and likely independent events, Midas cichlid generalist ancestors from the great Nicaraguan lakes colonized these newly formed crater lakes [14–18]. Midas cichlids have diversified sympatrically and allopatrically within and among the crater lakes and great lakes, sometimes on extremely short time scales [14–16].

Midas cichlids exhibit a conspicuous, sex-independent gold-dark color polymorphism and are named for the Greek mythology of King Midas, who turned everything he touched into gold. The gold morphs are found within many, but not all, populations and species in the Midas cichlid complex and their frequency is relatively low and variable (< 5–20 % of individuals in a given population) [17, 19]. All fishes start life phenotypically dark, but some time before sexual maturity the genetically gold individuals lose their dark pigmentation and become completely yellowish to orange in body and fin color [20, 21]. However, most individuals remain melanic, being dark-gray with vertical bars and dark fins (dark morph) (Fig. 1). This is a Mendelian trait determined by a two-allele locus with gold dominant over dark and almost complete penetrance [20, 21]. The gold phenotype is also correlated with social and competitive behavior [22–24]. For example, a recent analysis of two sympatric sister species in crater lake Xiloá found that, despite breeding in sympatry at the same depths and season, within both species the fishes paired highly color assortatively (95 % of *A. sagittae* pairs and 77 % of *A. xiloaensis* pairs were of the same color) [19]. A striking implication of this color-based sexual isolation was significant genetic differentiation between sympatric conspecific color morphs, on a level comparable to that found between recognized species [19].

The Midas cichlid color polymorphism is a genetically determined trait that is the basis of assortative mating [19], so theory predicts that incipient divergence by color may be possible if the color polymorphism has consequences for natural [9] or sexual selection [25, 26]. We tested the natural selection hypothesis through multivariate analyses of color-associated ecological (stable isotope ratios of carbon and nitrogen) and morphological divergence (body shape and jaw morphology) across replicate populations, coupled with a common garden experiment. Instances of parallel evolution, where similar phenotypes arise independently across different environments from a recent common ancestral form, provide strong evidence for natural selection in driving diversification [27, 28]. We focused on ten wild caught sympatric and allopatric color polymorphic populations (Fig. 1). Because the crater lakes were colonized from the ancestral great lake environment during only the last 100 to 6,000 years, and each crater lake population is genetically distinct from other populations [14], the crater lake populations can be considered “natural evolutionary experiments” with each being an independent replicate common garden experiment under natural conditions. The Nicaraguan great lakes and crater lakes Xiloá and Masaya each contain two sympatric color polymorphic Midas cichlid species, whereas only one color polymorphic species is currently known from crater lakes Apoyeque and Asososca Managua. The extent of genetic differentiation between species ranges from low and non-significant to high and significant [16, 19].

Across freshwater fishes in general [29, 30], including Midas cichlids [15, 16, 31], differentiation in body shape reflects differentiation in ecology, where deeper-bodied fishes are associated with a more benthic niche. Lower pharyngeal jaw (LPJ) robustness, shape and size is strongly associated with diet in cichlids: thicker, broader and heavier jaws with wider teeth have stronger crushing force and are associated with a benthic, durophagous, mollusk-rich diet (“molariform” jaw in Additional file 1: Figure S1b), while more gracile LPJ with finer teeth and less dense bone are found in fishes that eat a more piscivorous or planktivorous diet (“papilliform” jaw in Additional file 1: Figure S1b) and are associated with a more limnetic niche, e.g., [15, 16, 31–35]. Isotopic ratios of carbon (δ^13^C) and nitrogen (δ^15^N) reveal these long-term integrated signatures of diet. In aquatic systems, δ^13^C generally reflects the macro-habitat source (benthic vs. limnetic carbon origin), whereas δ^15^N corresponds to the trophic level and is indicative of consumer prey relationships [36] including extent of durophagy [16]. Studies on farmed fish have demonstrated that different diet compositions induce characteristic δ^13^C and δ^15^N signatures [37].

In the present study we assessed how color morphs differed in body shape and defining characteristics of LPJ morphology, such as shape and weight, from ten replicate populations. To infer whether the eco-morphological differentiation found between Midas cichlid color morphs corresponded to a systematic divergence in diet, we analyzed color-associated differences in δ^13^C and δ^15^N across the species complex. To synthesize the consistency of color morph divergence across populations, we conducted an evolutionary trajectory analysis [38] of multiple traits combined (i.e., test for parallelism of size and orientation across body shape, LPJ weight, δ^15^N, and δ^13^C). We also quantified the relative amount of shared and unique features of divergence for each trait using multivariate analysis of covariance (MANCOVA) [39].

According to theory a key criterion to facilitate sympatric divergence would be a genetic correlation of the color polymorphism that is the basis of assortative mating [19] and ecologically relevant traits [3, 8]. To test for such a genetic relationship, we conducted a common garden experiment under controlled laboratory conditions and assessed the eco-morphological variation between color morphs.

Based on these multiple lines of assessment, we present the first evidence for repeated ecologically relevant morphological divergences in sympatry based on a binary, sex-independent, and genetically determined color polymorphism.

## Results

### Ecological and morphological differentiation between color morphs

**Fig. 2:**
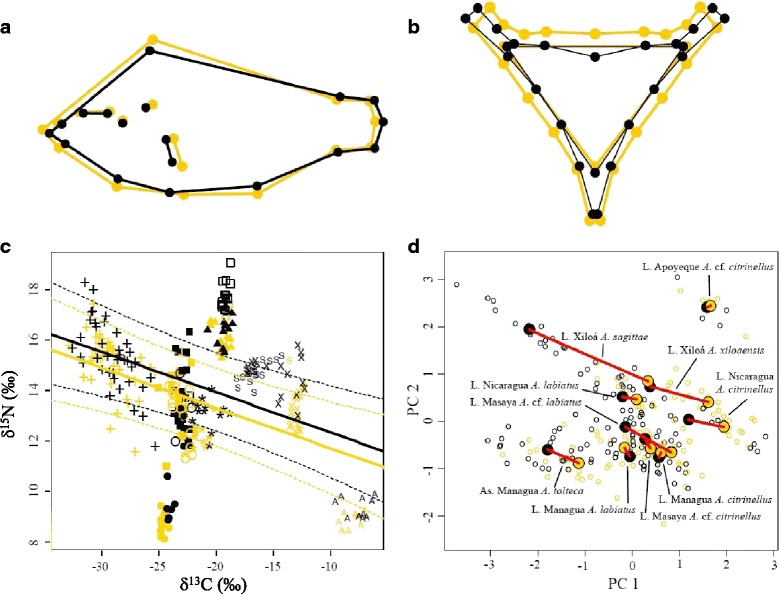
Ecological differentiation between Midas cichlid color morphs. **a** Body shape differs between gold and dark morphs (*n* = 1,177) when assessing the entire species complex (scale factor = 10). Gold morphs were deeper-bodied, had a larger head and a longer pectoral fin base, compared to dark morphs. **b** Lower pharyngeal jaw shape differs between gold morph and dark morph (scale factor = 10) (*n* = 465). Gold morphs tend to have a more robust lower pharyngeal jaw shape. **c** There is a systematic difference in stable isotope signatures of δ^15^N between color morphs across the ten populations (n = 298 individuals) suggesting long-term diet differentiation between morphs. Fitted regression lines of the linear mixed effects model are indicated by solid lines (dotted lines indicate the 95 % credible interval) for both color morph groups. Gold morphs fed at a relatively lower trophic level (δ^15^N) than dark morphs, independent of the population and while accounting for δ^13^C. Symbols indicate the groups as follows: *plus sign* As. Managua, *A. tolteca*; *filled square* L. Masaya, *A. cf. citrinellus*; O L. Masaya, *A. cf. labiatus*; *filled circle* L. Nicaragua, *A. citrinellus*; *asterisk* L. Nicaragua*, A. labiatus; filled triangle* L. Managua, *A. citrinellus*; *empty square* L. Managua, *A. labiatus*; S L. Xiloá, *A. sagittae*; x L. Xiloá, *A. xiloaensis*; A L. Apoyeque, *A. cf. citrinellus*). **d** Phenotypic trajectory analysis of combined trophic and eco-morphological traits (body depth index [BDI], lower pharyngeal jaw [LPJ] weight, and stable isotope ratios of δ^15^N and δ^13^C; *n* = 232) demonstrates parallel evolution of many gold-dark pairs across replicate populations. Most vectors of phenotypic difference (red lines) between the centroids of gold and dark morphs are parallel in orientation while being substantially different in length

Color morphs differed significantly from each other in body shape; the gold morph was generally deeper bodied and had a larger head compared to the dark morph. This was shown by a geometric morphometric analysis across the ten populations pooled (Procrustes distance = 0.01, Hotelling’s T^2^ = 248.06, *p* < 0.0001) (Fig. 2a). This pattern was generally consistent across populations examined individually; in most cases a discriminant function analysis clearly separated body shapes by color, although variable in extent and significance given the small sample sizes in some groups (Additional file 1: Table S1 and S2). While accounting for population-specific effects, gold morphs clearly had higher body depth indexes (BDI, a univariate proxy of overall body shape and corrected for allometry) than dark morphs (logistic regression: mean of posterior distribution = 15.75 [95 % credible interval = 9.53 to 21.84]; Additional file 1: Figure S2a, Additional file 1: Table S3).

Color morphs differed in LPJ shape (across populations pooled; Procrustes distance = 0.01, Hotelling’s T^2^ = 67.73, *p* < 0.0001; Fig. 2b), with gold morphs having more robust LPJs compared to dark morphs (Fig. 2b; Additional file 1: Figure S1). As with body shape, the differences in LPJ shape between color morphs in each population separately was variable in extent and significance (Additional file 1: Table S2). To accommodate the population effects, we conducted a binary logistic regression with size-corrected LPJ weight as a proxy of LPJ morphology [31]. This showed that gold morphs had consistently heavier, i.e., more robust and molariform [32], LPJs than dark morphs (mean of posterior distribution = 1.49 [95 % credible interval = 1.04 to 1.95], Additional file 1: Figure S2b, Additional file 1: Table S3), while accounting for population effects. As LPJs are well established indicators of diet in these and other cichlids [15, 16, 31], this significant weight difference suggests that gold morphs consume a more hard-shelled diet relative to dark morphs.

To test the link between ecologically relevant morphology and trophic level, we examined the difference in δ^15^N between color morphs while accounting for δ^13^C variability across the populations [40]. We found that gold morphs were consistently lower than dark morphs in δ^15^N (model coefficient: −0.63 ‰ [95 % credible interval = −0.86 ‰ to −0.39 ‰]) (Fig. 2c). This Stable isotope differences suggest that sympatric gold and dark color morphs consistently exploit a slightly different trophic position [36] throughout the species complex. This is additionally supported by the consistent pattern of ecologically relevant divergence in body shape and LPJ size and shape.

### Parallel and non-parallel patterns of divergence

To synthesize across all traits and test the consistency of ecologically relevant divergence in a multivariate framework, we conducted a phenotypic trajectories analysis of all traits combined (BDI, LPJ weight, isotopic signatures of δ^15^N and δ^13^C) [38]. Trajectory path lengths, which describe the amount of phenotypic evolution across all traits between sympatric color morph pairs, differed overall between gold and dark across the ten morph pairs (Δ*d* = 0.48, *p* = 0.005). This suggests different magnitudes of divergence across replicate color morph pairs (Fig. 2d, Additional file 1: Table S4), which would be expected given the considerable variation in population age and demography across replicates [14, 17, 18]. The trajectory path orientations, which describe trait co-variation through phenotypic space, appeared substantially parallel for most morph pairs (Fig. 2d) and the hypothesis of parallelism was never rejected statistically in any pairwise comparison (Additional file 1: Table S4). However the null hypothesis of parallel orientation across all replicates was rejected (*θ* = 1625.74, *p* = 0.001), likely due to three color morph pairs with the shortest vector lengths (*A. citrinellus* and *A. labiatus* from Lake Managua, *A.* cf*. citrinellus* from Lake Apoyeque) that deviated in vector direction from the others (Fig. 2d). Nonetheless, with the exception of those three populations, there is a consistent co-variation in phenotypic space between color morph pairs (orientation along PC1 and PC2) across population replicates (Fig. 2d).

We further assessed the shared and unique aspects of color morph divergence in each trait using MANCOVA [39] for each trait separately. Predictably, because each color polymorphic population differs in age and demographic history [14], the largest amount of trait variance in LPJ size, δ^15^N and BDI was due to differences in evolutionary history of the morph pairs (”evolutionary replicate”; Table 1). However, we found that in all cases the effect of color (percent variance explained) exceeded any unique population-specific variation between color morphs (“color x evolutionary replicate”; Table 1). Together, the MANCOVA and the evolutionary trajectory analysis evidence a relatively consistent parallelism of ecological divergence across most replicates of sympatric gold-dark morph pairs.

**Table 1:** Shared and unique aspects of divergence in ecologically relevant traits

Test for	Trait	Factor	F	df	P	Partial variance explained (%)
Shared divergence among color morphs	BDI	Color	161.55	1	<.0001	10.8
Impact of evolutionary history		Evolutionary replicate	223.38	9	<.0001	60.1
Unique divergence in the morph pairs		Color x evolutionary replicate	14.86	9	<.0001	9.1
Shared divergence among color morphs	LPJ weight	Color	137.33	1	<.0001	24.0
Impact of evolutionary history		Evolutionary replicate	66.98	9	<.0001	58.0
Unique divergence in the morph pairs		Color x evolutionary replicate	4.37	9	<.0001	8.3
Shared divergence among color morphs	δ^15^N	Color	64.16	1	<.0001	18.8
Impact of evolutionary history		Evolutionary replicate	159.09	9	<.0001	83.7
Unique divergence in the morph pairs		Color x evolutionary replicate	2.37	9	0.01	7.1

MANCOVA results and partial η^2^-values for the relationship between lower pharyngeal jaw (LPJ) weight, δ^15^N, and body depth index (BDI) across all ten color polymorphic populations. In all ecological traits a substantial amount of the variance is due to differences in evolutionary history (factor “evolutionary replicate”). However, across all traits a larger portion of the variance is explained by color (factor "color") rather than population-specific effects (factor “color x evolutionary replicate”). This corroborates our previous findings of a relatively parallel ecological divergence based on color across the species complex

### Common garden experiment

**Fig. 3:**
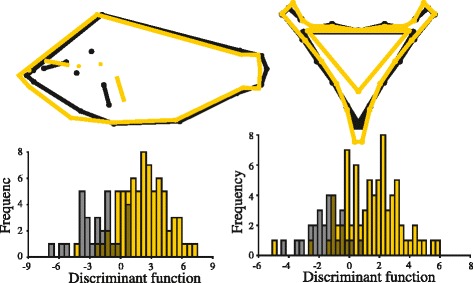
Genetically determined eco-morphological differentiation between color morphs. There is significant body shape and lower pharyngeal jaw (LPJ) shape differentiation between sibling color morphs raised in the same environment (*n* = 79 gold, 28 dark). The direction of body shape and LPJ shape differentiation was similar to that observed in nature (Fig. 2a and b); gold siblings had larger heads and rather robust LPJ morphologies compared to dark siblings. Scale factor = 10

To test whether these consistent differences in eco-morphology had a genetic basis, we raised gold and dark sibs (F2 cross of wild caught parents) under identical conditions in the laboratory from hatching to adult-sized. We found that body shape differed significantly between these laboratory-raised gold and dark sibling fishes (Procrustes distances^Body shape^ = 0.010, Hotelling’s T^2^ = 92.58, *p* = 0.006). Further, we found that LPJ shape also differed significantly between sibs of different colors (Procrustes distances^LPJ shape^ = 0.014, Hotelling’s T^2^ = 59.36, *p* = 0.007), despite being raised on identical foods. The eco-morphological differentiation was of the same pattern and direction, though to a lesser extent, as we found in the wild populations; laboratory-reared gold individuals had a larger head and sturdier pharyngeal jaws than their dark morph sibs (Fig. 3). Our data lend support to the hypothesis that the eco-morphological variation we identified between sympatric conspecific color morphs in the ten wild populations is genetically correlated with color.

## Discussion

### Parallel and non-parallel divergence based on color

Across replicate populations of Midas cichlids, including populations from completely isolated crater lakes derived from independent colonizations [14, 18], we identified considerable and in most cases repeated ecological differentiation between sympatric gold and dark color morphs. Further, this differentiation follows an ecological and evolutionary pattern that is consistent across the species complex overall. This is evident in ecological and morphological traits such as body shape (Fig. 2a, Additional file 1: Table S2), LPJ shape (Fig. 2b, Additional file 1: Table S2), and stable isotope signatures of δ^15^N (Fig. 2c, Additional file 1: Table S2), including those statistical models that take the binary nature of the trait and the population effects into account (Additional file 1: Figure S2a, b; Fig. 2c). Combining the eco-morphological traits into a single trajectory analysis demonstrated the consistency of the divergence pattern, with color morph pairs in most populations separating along the primary and secondary axis of variation (Fig. 2d). This intraspecific differentiation is largely parallel across most populations. This suggests a prominent role of natural selection in bringing about the observed differences [29]. As far as we are aware, this is the most replicated and consistent ecological and genetic differentiation that has been found to associate with a sex-independent binary color polymorphism [3].

The observed color morph divergence occurs along a benthic-limnetic ecological axis associated with durophagy. The pattern of this eco-morphological divergence is comparable to the divergence along the benthic-limnetic axis that is ubiquitous in freshwater fishes [16, 29, 30, 41]. The varying extent and in some cases deviating directions of color morph divergence are likely to reflect different stages of ecological divergence across the species complex, due not only to evolutionary histories but also to unique environmental differences across lakes in regard to size, depth and ecological opportunity [17, 42, 43]. For example, deep, clear-watered, and young crater lake environments strongly differ from the turbid and shallow waters of the Nicaraguan great lakes (reviewed in [17, 43]) and, therefore, crater lake Midas cichlids inhabit substantially different environments than the ancestral populations. Further, it is conceivable that environmental differences, e.g., in turbidity, cause different strengths of color assortative mating across morph pair replicates from different lakes [6, 13], though this remains to be tested. Such factors may explain why eco-morphological differentiation between color morphs tends to be more pronounced in the crater lake populations than in the great lake populations for body shape (mean Procrustes distances^Body shape^ in crater lakes = 0.019 vs great lakes = 0.016) and for LPJ shape (mean Procrustes distances^LPJ shape^ in crater lakes = 0.02 vs great lakes = 0.017, Additional file 1: Table S2). This pattern is in agreement with population genetic analyses that found significant differentiation between sympatric color morphs in a crater lake species [19] yet little or no differentiation in the great lakes species [44]. Similarly, genetic differentiation is higher between Midas species in the crater lakes than between species in the great lakes [14, 16, 40]. It may well be that selection pressures and differences in habitat use between the two color morphs, possibly caused by differential predation by aquatic and/or aerial predators [45, 46], are stronger in the clear-watered crater lake than in the murky great lake environment [13]. However, despite the dramatic differences in the physical and ecological environments of the different lakes, ecological and morphological divergence across color morph pairs is highly consistent.

### A mechanism to facilitate divergence based on color?

The persistent eco-morphological differentiation in body shape and LPJ morphology of the ten investigated natural populations and the gold and dark common garden siblings suggests that the color polymorphism has potentially direct, genetically determined, ecological consequences [32]. Further experiments are needed, either to confirm the genetic effects under controlled laboratory conditions or through tracking the specific genetic basis of color in wild populations. If our hypothesis of a genetic correlation of the mating cue “color” and ecology is confirmed, the genetic correlation is likely to play a major role in the maintenance of the color polymorphism, and possibly facilitates sympatric incipient diversification [19, 44] by reducing ecological competition. The common garden experiment suggested a sizable genetic component of those color-associated eco-morphological differences, which provides a genetic mechanism to facilitate sympatric ecological divergence based on the color polymorphism [3, 6, 8]. The coexistence of color morphs may even suggest that the morphs occupy different fitness peaks in an adaptive landscape [10, 29, 47] maintained by assortative mating [19]. However, although we suggest there is a genetic correlation of color and ecology in Midas cichlids, it so far remains unclear whether the color polymorphism constitutes pleiotropy, a single-locus “magic trait” [10] or tight physical linkage of the color gene and the genes that bring about eco-morphological differentiation [8]. Furthermore, it still remains to be determined whether the color polymorphism originated *de novo* within each evolutionary replicate or whether it was brought into the crater lakes with their founding population. The prevalence of the color polymorphism in this species complex (found in most but not all lakes and species) strongly suggests that this is an ancestral trait, but its genetic architecture and evolutionary history remain to be determined.

Despite the divergence in ecological phenotypes between sympatric conspecific color morphs we have no evidence that the color polymorphism is a mechanism for speciation in this young system. In no case has full speciation occurred in Midas cichlids based on color, despite assortative mating according to color [15, 16, 48]. Ecological diversification in Midas cichlids is, however, abundantly associated with phenotypic traits other than color as demonstrated by the benthic-limnetic flock in crater lake Apoyo, with its six described species that all lack the color polymorphism [16], or the thin-lipped/thick-lipped morphs found in some lakes [40, 48]. In fact, it is fascinating that this color polymorphism is repeatedly found within species that have diversified in sympatry along the benthic-limnetic axis (e.g., in crater lake Xiloa [19]), yet is never itself the focus of speciation – that is, there is no species in the complex that is strictly gold. Thus, it remains unclear whether divergence based on the color polymorphism is simply slower than divergence based on traits other than color, or if the color polymorphism does not result in speciation because of genetic constraints or due to ecological reasons. The highly replicated color polymorphism across at least ten pairs, and their consistent eco-morphological differentiation in sympatry, strongly suggests this is an ancestral polymorphism maintained in the populations through selection and assortative mating.

## Conclusions

Here we have documented a robust example of a sex-independent color polymorphism being associated with ecological divergence in a replicate manner, as well as suggesting a genetic correlation of that color polymorphism with eco-morphology. This suggests a potential, but yet untested, role of color polymorphisms in promoting evolutionary diversification [19]. Our results demonstrate the relevance of a color polymorphism in maintaining sympatric ecological divergence within and across populations and species.

## Methods

### Specimen collection

Midas cichlids were sampled from the great lakes and crater lakes of Nicaragua by gill-netting or harpooning between 2001 and 2012 (Additional file 1: Table S1) to collect 1,354 individuals from ten populations. In the field, standardized photographs of the left body side of each fish were taken from directly above. All specimens were taken as vouchers (head or whole body) and stored in 70 % ethanol.

### Assessment of eco-morphology and diet

#### Body depth index and body shape

We tested if sympatric, conspecific color morphs of Midas cichlids differed in body shape across the ten populations (Fig. 1) using geometric morphometric analyses (Additional file 1: Figure S1, Additional file 1: Table S1). Eighteen landmarks (LM) describing body shape of 1,354 individuals were digitized from standardized photographs in tpsDig v. 2.16 [49] by a single investigator (Additional file 1: Figure S1, landmarks modified from [40]). Body Depth Index (BDI) is the relative fraction of body depth (distance LM 6 to LM 9 in Additional file 1: Figure S1) divided by standard length (distance LM 1 to LM 15 in Additional file 1: Figure S1). BDI was calculated from inter-landmark distances that were obtained in PAST v. 2.16 [50] for each individual. BDI is a proxy of overall body shape and was therefore used in the logistic regression (below).

The body shape data, but not BDI, showed some temporal sampling effects. Therefore, a slightly reduced sample was used for the multivariate analyses of shape (Additional file 1: Table S1, *n* = 1,177). Analyses were performed in MorphoJ v. 1.05c [51], following a previous study on Midas cichlid body shape differentiation [17]. Body shape data exhibited significant allometric effects (5.04 % of shape variation explained by centroid size; *p* < 0.0001), thus allometry-corrected shape data (regression residuals) were used in downstream analyses. Discriminant function analysis (DFA) with cross-validation was used to test for significant differentiation between mean color morph body shapes within each population (Additional file 1: Table S2).

#### Lower pharyngeal jaw size and shape

To quantify the LPJ difference between color morphs, we conducted a multivariate analysis of shape using geometric morphometrics and measured LPJ weight (Additional file 1: Figure S1, Additional file 1: Table S1). Standardized photographs were taken of extracted and cleaned lower pharyngeal jaws (Additional file 1: Table S1, *n* = 465) from directly above using a digital camera. Twenty-four homologous landmarks, consisting of 12 fixed and 12 semi-landmarks were defined that describe the external LPJ shape along with the dentigerous area (Additional file 1: Figure S1). Digitization was done using tpsDig v. 2.16 [49] by a single investigator from the specimen photographs. Semi-landmarks were slid in tpsRelw v. 1.49 [52] in orthogonal projection mode with 10 iterations. Slid semi-landmarks were treated as true homologous landmarks in MorphoJ v. 1.05c [51]. Object symmetry was taken into account and the symmetric component of shape variation only was considered as our trait of interest [53]. A correction for allometric effects on LPJ shape was performed by regressing Procrustes coordinates on LPJ centroid size (4.31 % of shape variation explained by centroid size; *p* < 0.0001). Regression residuals were used in downstream analyses that were conducted analogous to body shape analyses. In a complementary approach jaws were weighed to the nearest mg using a digital scale. Cube root normalization was applied to allometry-corrected LPJ weight. LPJ weight is representative of overall LPJ morphology [31] and was therefore used in the logistic regression analysis (below).

#### Stable isotope analysis

A small piece of muscle tissue was extracted from dorsal musculature of 298 ethanol preserved specimens (Additional file 1: Table S1) and dried for about 48 hours at 55 °C. Samples were ground in individual sealed tubes and a 1.0–1.5 mg subsample was weighed. Analyses were done by gas chromatography combustion isotope ratio mass spectrometry (GC-C-IRMS) at the Limnological Institute of the University of Konstanz. The δ^13^C-values were corrected for lipid content [54]. Isotopic differentiation between color morphs within each population was investigated in a comparative framework as described below.

### Comparative data analysis of BDI, LPJ weight and stable isotopes

#### Binary logistic regression on eco-morphology

Pre-analyses suggested a major and population independent axis of differentiation between color morphs. To test for a consistent eco-morphological differentiation between color morphs across the species complex, logistic regression with a binominal error distribution was used and conducted in R v. 2.15.1 [55]. Being gold (“1”) or not (“0”≙ dark) was set as response variable and allometry-corrected BDI or LPJ weight was set as explanatory variable. To test for an overall effect across the species complex, each population was modeled as a random factor and a flat prior distribution was used. The *glmer-*function (package “*lme4*” [56]) was used to fit the models. The *sim-*function (package “*arm*” [57]) was used for simulation of 5,000 values from the posterior distribution of the model parameters. Inference was drawn based on the 95 % credible interval (CrI), which is the Bayesian analog to confidence interval. Conventionally, if zero is not included in the Bayesian 95 % CrI, an effect is considered to be “clear” [58]. Logistic regression coefficients were interpreted following Gelman and Hills’ “divide by 4 rule” [57] as predicted probabilities of the model outcome. Please see Additional file 1: Figure S2 for a practical example of how to apply this rule.

#### Linear mixed effects model on stable isotope signatures

To test for a systematic differentiation between color morphs in the trophic level δ^15^N while accounting for the correlation with δ^13^C [31] and accommodating the environmental variation across lakes, we designed a linear mixed effects model. As fixed effects for the model we entered “color morph” and “δ^13^C” (without interaction) and each morph pair (“evolutionary replicate”) was considered as a random effect under the assumption of a common slope for each morph pair. The *lmer-*function (package “*lme4*” [56]) was used to fit the models. The *sim-*function (package “*arm*” [57]) was used for simulation of 5,000 values from the posterior distribution of the model parameters. Visual inspection of residual plots revealed that the model assumptions (homoscedasticity, normality, etc.) were adequately met (Additional file 1: Figure S3). Inference was drawn, based on the 95 % CrI as described above for eco-morphology.

#### Evolutionary trajectory analysis

To synthesize the consistency of color morph divergence across replicates, we conducted an evolutionary trajectory analysis [38] of all traits (i.e., parallelism of size and orientation across BDI, LPJ weight, δ^15^N, and δ^13^C). Analyses were conducted on all ten morph pairs simultaneously (factor 1 = morph pair, factor 2 = gold or dark). Evolutionary trajectories were analyzed by principal component analysis on z-standardized variables following a generalized linear model to calculate color morph centroids and vectors between sympatric color morph groups, as described in [38, 59]. The differences in length and orientation across the ten vectors were compared statistically with the “residual randomization method” [59] employing 9,999 permutations.

#### Shared and unique features of divergence

To quantify the relative amount of shared and unique features of divergence across the species complex, a MANCOVA analysis was performed according to the method in [39]. MANCOVA was used to determine the relative effect sizes of shared divergence among color morphs (color), morph pair history (morph pair) and unique aspects of divergence among morph-pairs in the different morph-pairs (color:morph pair) on the ecological variables BDI, LPJ weight and δ^15^N. Partial η^2^-values were calculated for those factors and indicate the explanatory ability of a factor relative to unexplained variation.

### Assessment of genetic correlation of the color polymorphism and eco-morphology

To test for such a genetic relationship between the color polymorphism and ecologically relevant traits, we conducted a common garden experiment under controlled laboratory conditions using sibling fish derived from a Midas cichlid F2-intercross heterozygous for the gold locus. This laboratory strain was collected by George Barlow (University of California, Berkeley) in the early 1970s and derives from Lake Masaya [60]. We analyzed eco-morphology in a manner analogous to the wild-caught samples. In this common garden approach, 107 Midas cichlid sibling offspring of an F2-intercross (heterozygous for the gold-locus) between gold/dark F1-hybrids [20] were raised together in the same tank throughout their life, first in a 600 L tank in the animal research facility of the University of Konstanz and later in a large 10,000 L “mesocosm” in the Limnological Institute of the University of Konstanz. Fish were fed to excess daily with commercial pellet food. Fishes were sacrificed at about 20 months of age after exceeding a standard length of ~11 cm. This size range is similar to that of wild-caught adult fish in our field collection (see Additional file 1: Table S3). Phenotypically, 68 individuals were gold and 39 individuals were dark. Two microsatellite loci that are in strong linkage disequilibrium with the gold*-*locus (F. Henning, unpublished data) were amplified and genotyped for all dark individuals, as well as for a single gold individual as a control, using established conditions. This was done to genetically discriminate if any phenotypically dark individuals were actually untransformed gold individuals. Due to the Mendelian inheritance of gold color, the gold to dark offspring ratio of a mating involving two heterozygous gold parents should be 3:1. As expected, we found 79 genetically gold and 28 genetically dark morphs in our cross. Most genetically gold individuals were already transformed or still in transition. However*,* 11 genetically gold individuals had not yet transitioned from dark and could only be identified genetically. All fish were processed the same day and eco-morphological color morph differentiation has been assessed as described above for the field specimens (gold:dark samples sizes: body shape = 74:28; LPJ shape = 75:27).

## Additional file

Additional file 1: **Supplementary Material (all files combined). Figure S1.** Definition of eco-morphological measurements. **Figure S2.** Logistic regression: frequency distributions of the linear predictor for gold and dark morphs. **Figure S3.** Residual analysis of stable isotope data. **Table S1.** Sample sizes of color morphs used for each analysis. **Table S2.** Geometric morphometrics: Pair-wise morphological differentiation between Midas cichlid color morphs. **Table S3.** Overview of color-associated divergence in eco-morphology and stable isotope ratios. **Table S4.** Evolutionary trajectory analysis. (PDF 1003 kb)
