# Supplementary material for: Sympatric ecological divergence associated with a color polymorphism
Source: BMC Biol. 2015 Oct 5;13:82. doi: 10.1186/s12915-015-0192-7 (PMC4594650; doi:10.1186/s12915-015-0192-7)
Supplement: Supplementary file 1 — Supplementary Material (all files combined). Figure S1. Definition of eco-morphological measurements. Figure S2. Logistic regression: frequency distributions of the linear predictor for gold and dark morphs. Figure S3. Residual analysis of stable isotope data. Table S1. Sample sizes of color morphs used for each analysis. Table S2. Geometric morphometrics: Pair-wise morphological differentiation between Midas cichlid color morphs. Table S3. Overview of color-associated divergence in eco-morphology and stable isotope ratios. Table S4. Evolutionary trajectory analysis. (PDF 1003 kb) [file 12915_2015_192_MOESM1_ESM.pdf]

Additional Files

Figure S1. Definition of eco-morphological measurements. a, Anatomical description of body landmarks: LM 1 = tip of snout; LM 2 = most posterior point of the lips; LM 3, 4 = most anterior and posterior margin of the eye; LM 5 = intersection of operculum and ventral body outline; LM 6 = anterior base of dorsal fin; LM 7, 8 = dorsal and ventral base of pectoral fin; LM 9 = anterior base of pelvic fin; LM 10, 11 = anterior and posterior base of anal fin; LM 12 = posterior base of dorsal fin; LM 13, 14 = dorsal and ventral base of caudal fin; LM 15 = midpoint of the caudal fin origin; LM 16 = nostril; LM 17 = dorsal end of pre-operculum; LM 18 = dorso-caudal origin of the operculum. b, Anatomical description of LPJ landmarks along with representative examples of "molariform" and "papilliform" LPJ morphologies: LM 1, 2 & LM 10, 11= tips of posterior and lateral processes of upper horn; LM 3, 9 = points of highest curvature in upper horn base; LM 4, 8 = points of closest intersection between horn base and dentition area; LM 5, 7 = points of highest curvature near the mid-point of the two adjacent landmarks; LM 6 = posterior-most point of lower pharyngeal jaw suture; LM 12-15 & 18-21 = points of highest curvature near the mid-point of the two adjacent landmarks; LM 16, 17 = anterior tips of lower horn processes; LM 22, 23 = most posterior teeth of the "external line" of the dentition area; LM 24 = point where the suture meets the dentition area.

Figure S1.

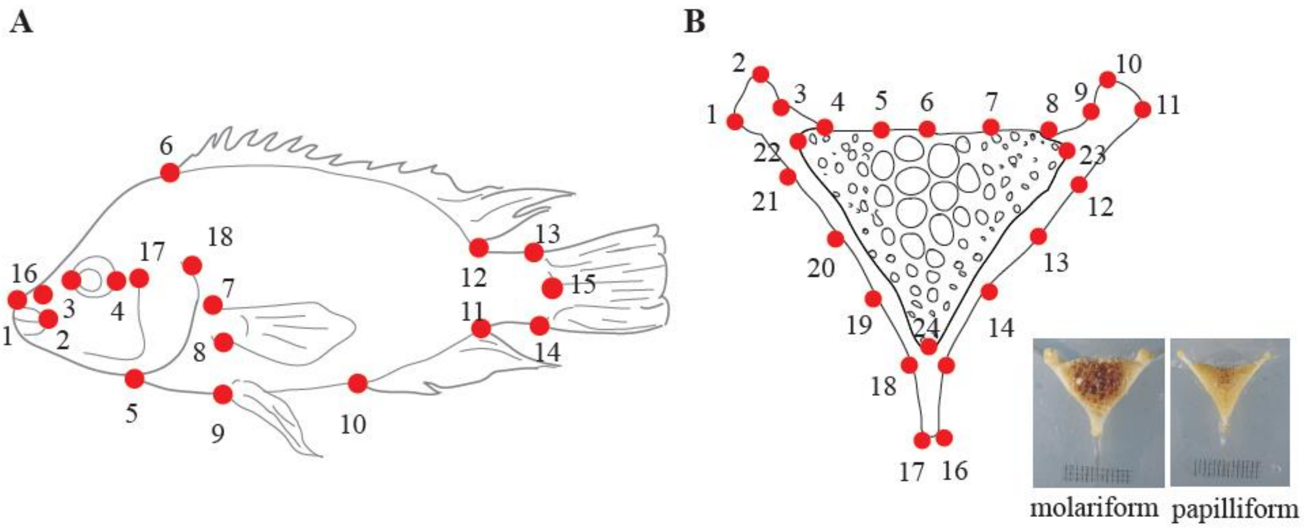

Figure S2. Logistic regression: Frequency distributions of the linear predictor for gold and dark morphs. a, Body Depth Index and b, LPJ weight. The histograms (upper panels) indicate absolute frequency of individuals of each color morph (y-axis), whereas the density distributions (bottom panels) are corrected for unequal sample sizes. The linear predictor (x-axis) is a linear function that defines the relationship between the dependent (i.e. gold/dark) and the explanatory variables (i.e. BDI or LPJ weight, both corrected for allometry). The likelihood of a given individual being a gold morph, as predicted by the model and reflected in the linear predictor, clearly increased with higher values of BDI and LPJ weight. The linear predictor for BDI was -0.99 +15.75*BDI and the one for LPJ weight was -0.45 + 1.49*LPJ weight. Gelman and Hill's "divide by 4 rule" [57] allows to interpret logistic regression coefficients in terms of the predicted probabilities of the model outcome (slope for BDI-model = 15.75; slope for LPJ weight-model 1.49). Practically, when considering a shift in increasing BDI of ~0.1 (BDI ranges from -0.1 to 0.09, spanning 0.19 units) the probability of being gold increased by maximally 39.4 %. When considering a relative shift of LPJ weight of increasing 1 unit (LPJ weight ranges from -1.93 to 2.23, spanning 4.16 units) the probability of being gold increased by maximally 37.3 %.

Figure S2.

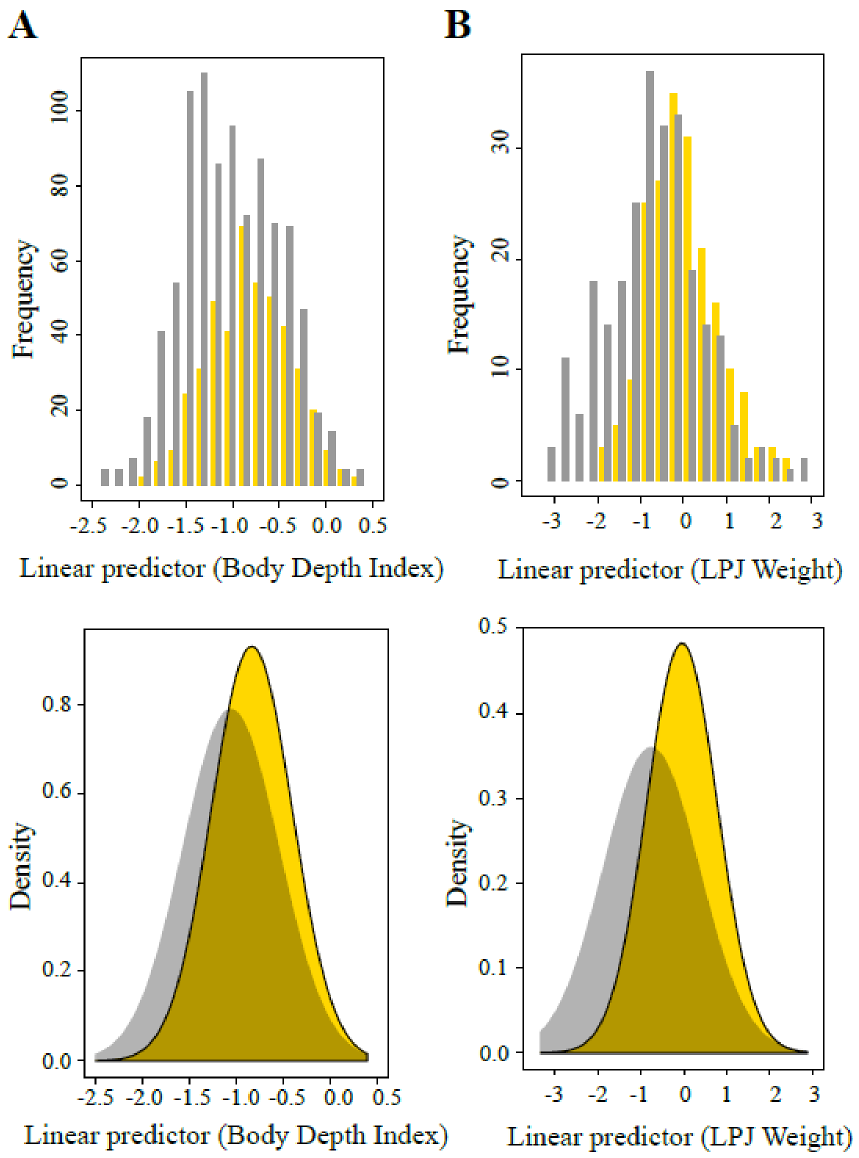
A
B
Frequency
Linear predictor (Body Depth Index)
Linear predictor (LPJ Weight)
Density
Linear predictor (Body Depth Index)
Linear predictor (LPJ Weight)

Figure S3. Residual analysis of stable isotope data. The assumptions of the linear mixed effects model dealing with color morph differentiation in stable isotopes were visually assessed. a, Plot of residuals vs. fitted values to assess possible deviations of the residuals' mean from zero. b, Q-Q-plot of residuals, to assess whether the residuals conform to normality. c, Plot of residual variance vs. fitted values to evaluate variance homogeneity. d, Q-Q-plot of random effects to assess whether random effects are normally distributed after implementation into the model.

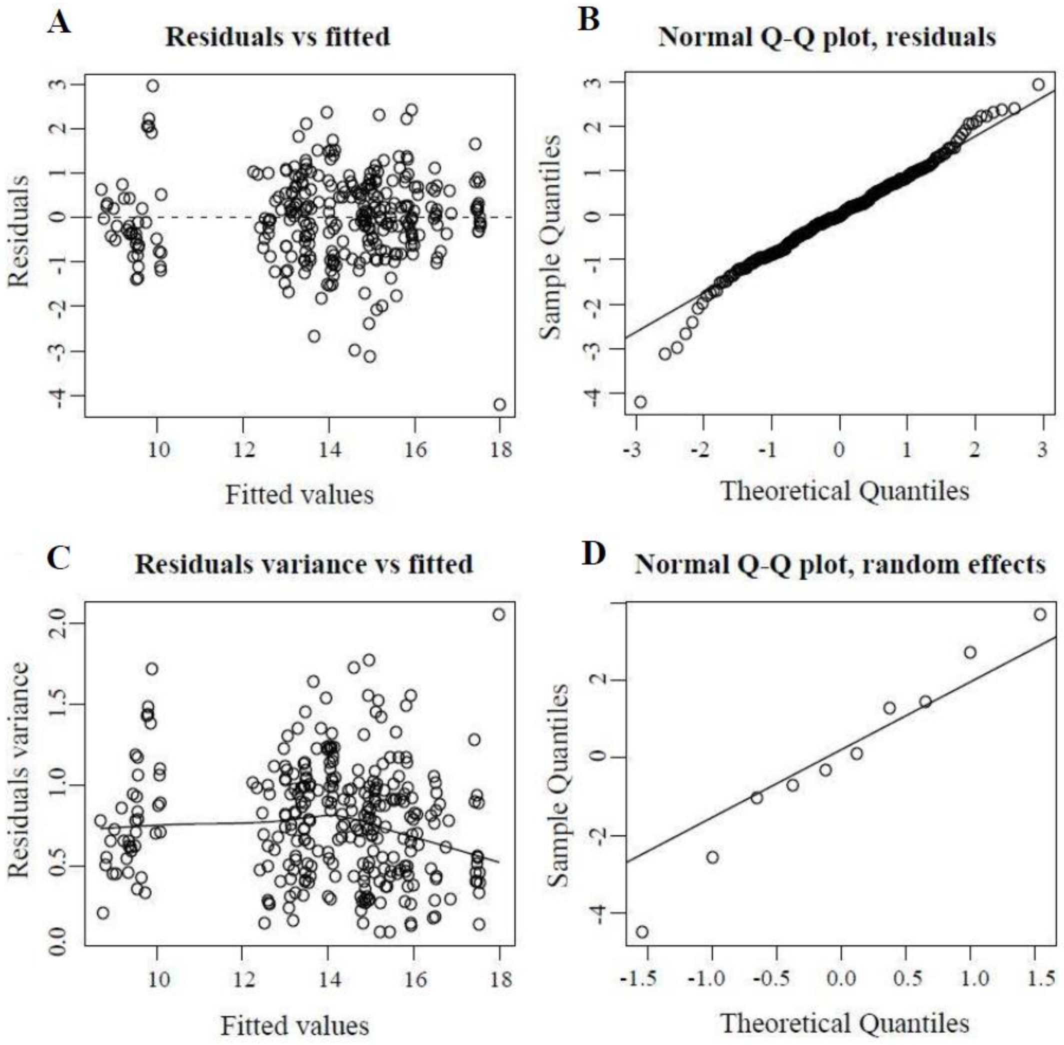

Table S1. Sample sizes of color morphs used for each analysis.

Lake	Species	Body shape ($n = 1{,}117$)		BDI ($n = 1{,}354$)		LPJ shape ($n = 465$)		Stable isotopes ($n = 298$)		Evol. trajectory ($n = 232$)	
		n gold	*n* dark	*n* gold	*n* dark	*n* gold	*n* dark	*n* gold	*n* dark	*n* gold	*n* dark
As. Managua	*A. tolteca*	51	138	52	232	43	54	36	38	28	26
Apoyeque	*A.* cf. *citrinellus*	7	25	7	107	7	19	8	11	7	6
Masaya	*A.* cf. *citrinellus*	16	38	16	38	15	33	13	20	10	15
	A. cf. *labiatus*	13	2	13	2	8	2	17	3	8	2
Xiloá	*A. sagittae*	6	133	6	133	4	39	2	20	2	14
	A. xiloaensis	67	77	67	77	31	21	18	16	14	10
Managua	*A. citrinellus*	60	114	60	114	24	21	16	14	15	13
	A. labiatus	4	32	4	32	4	13	2	16	2	13
Nicaragua	*A. citrinellus*	104	120	104	120	32	30	10	11	10	11
	A. labiatus	114	56	114	56	34	31	14	13	13	13

Table S2. Pair-wise morphological differentiation between Midas cichlid color morphs. The outcome of discriminant function analyses of body shape and lower pharyngeal jaw morphology is shown for each of the ten morph pairs. The degree of body and pharyngeal jaw shape differentiation is indicated by Procrustes distances.

		**Body shape**			**LPJ shape**		
**Lake**	**Species**	**Proc. dist.**	**Hotel. T^2**	***p*-value**	**Proc. dist.**	**Hotel. T^2**	***p*-value**
As. Managua	*A. tolteca*	0.011	118.78	<0.0001	0.012	80.58	<0.0001
Apoyeque	*A.* cf. *citrinellus*	0.022	6002.65	0.304	0.019	368.74	0.328
Masaya	*A.* cf. *citrinellus*	0.013	116.09	0.182	0.007	20.85	0.931
	A. cf. *labiatus*	0.02	79.69	0.766	0.036	20.34	0.174
Xiloá	*A. sagittae*	0.023	98.22	0.002	0.025	49.35	0.414
	A. xiloaensis	0.025	265.03	<0.0001	0.022	167.34	<0.0001
Managua	*A. citrinellus*	0.01	67.99	0.01	0.037	113.38	0.014
	A. labiatus	0.03	121.04	0.954	0.016	85.6	0.886
Nicaragua	*A. citrinellus*	0.012	189.56	<0.0001	0.006	24.23	0.817
	A. labiatus	0.01	96.25	<0.0001	0.008	36.99	0.353

Table S3. Overview of color-associated divergence in eco-morphology and stable isotope ecology. The table shows mean regression residuals of BDI and LPJ weight after allometry correction, $\delta^{15}N$ as well as standard length for each group. Boldface indicates the color morph group with relatively higher values within each morph pair. The average gold morph in each morph pair generally exhibited higher BDI (7/10) and LPJ values (9/10), even after correcting for size and allometric effects. In 9 out of 10 morph pairs the gold morph was at a relatively lower trophic level than the dark morph as inferred from $\delta^{15}N$.

		BDI [mean]		LPJ weight [mean]		$\delta^{15}N$ [‰]		Standard length [mm]	
Lake	Species	gold	dark	gold	dark	gold	dark	gold	dark
As. Managua	*A. tolteca*	**-0.005**	-0.026	**-0.002**	-0.386	15.09	**15.42**	100.6	**113.2**
Apoyeque	*A.* cf. *citrinellus*	0.002	**0.005**	**0.178**	0.017	8.9	**9.36**	99.3	**102.3**
Masaya	*A.* cf. *citrinellus*	**0.032**	0.024	0.058	**0.086**	**13.9**	13.81	90.2	**106.3**
	A. cf. *labiatus*	**0.039**	0.012	**0.264**	-0.032	13.19	**13.36**	78.5	**92.2**
Xiloá	*A. sagittae*	**-0.017**	-0.041	**-0.330**	-1.217	14.51	**14.78**	**178.0**	168.6
	A. xiloaensis	**0.021**	-0.005	**0.533**	-0.338	13.22	**14.62**	158.0	**158.1**
Managua	*A. citrinellus*	**0.042**	0.036	**0.263**	0.131	15.93	**16.53**	**165.3**	160.0
	A. labiatus	**0.007**	-0.002	**0.460**	0.314	17.18	**17.51**	112.8	**154.9**
Nicaragua	*A. citrinellus*	0.011	**0.023**	**1.078**	0.785	8.72	**10.61**	**154.9**	148.6
	A. labiatus	-0.014	**-0.009**	**-0.250**	-0.311	12.43	**13.15**	136.4	**146.4**

Table S4. Evolutionary trajectory analysis. The evolutionary trajectory analysis is based on the ecological variables BDI, LPJ weight as well as the isotopic signatures of $\delta^{15}N$ and $\delta^{13}C$. The table shows absolute values (above the diagonal) and associated *p*-values (below the diagonal) for pair-wise differences in trajectory size and orientation. Bold font indicates *p*-values that differ statistically (< 0.05; no correction for multiple tests). Across all comparisons combined, length (Δd = 0.48, *p* = 0.005) and orientation (θ = 1625.74, *p* = 0.001) differ though there is a large component of trajectories being parallel across many morph pairs (Fig. 2d).

		Trajectory size									
		Apoyeque *A.* cf. *citrinellus*	As. Managua *A. tolteca*	Managua *A. citrinellus*	Managua *A. labiatus*	Masaya *A.* cf. *citrinellus*	Masaya *A.* cf. *labiatus*	Nicaragua *A. citrinellus*	Nicaragua *A. labiatus*	Xiloá *A. sagittae*	Xiloá *A. xiloaensis*
	Apoyeque *A.* cf. *citrinellus*		0.01	0.50	0.01	0.45	0.41	0.77	0.26	2.04	0.59
	As. Managua *A. tolteca*	0.980		0.49	0.00	0.44	0.42	0.78	0.25	2.05	0.60
	Managua *A. citrinellus*	0.249	0.128		0.483	0.05	0.91	1.26	0.24	2.54	1.09
	Managua *A. labiatus*	0.981	0.993	0.338		0.43	0.43	0.78	0.24	2.06	0.61
***p*-value**	Masaya *A.* cf. *citrinellus*	0.307	0.195	0.898	0.388		0.86	1.21	0.19	2.49	1.04
	Masaya *A.* cf. *labiatus*	0.449	0.375	0.107	0.467	0.121		0.35	0.67	1.63	0.18
	Nicaragua *A. citrinellus*	0.092	**0.031**	**0.001**	0.138	**0.002**	0.489		1.02	1.28	0.17
	Nicaragua *A. labiatus*	0.563	0.450	0.515	0.634	0.614	0.205	**0.008**		2.30	0.85
	Xiloá *A. sagittae*	**0.001**	**0.001**	**0.000**	**0.001**	**0.000**	**0.012**	**0.019**	**0.000**		1.45
	Xiloá *A. xiloaensis*	0.185	0.079	**0.004**	0.239	**0.006**	0.720	0.673	**0.025**	**0.010**	

		Trajectory direction [°]									
		Apoyeque *A.* cf. *citrinellus*	As. Managua *A. tolteca*	Managua *A. citrinellus*	Managua *A. labiatus*	Masaya *A.* cf. *citrinellus*	Masaya *A.* cf. *labiatus*	Nicaragua *A. citrinellus*	Nicaragua *A. labiatus*	Xiloá *A. sagittae*	Xiloá *A. xiloaensis*
	Apoyeque *A.* cf. *citrinellus*		106.24	109.29	158.57	127.92	81.52	28.68	135.24	95.24	112.32
	As. Managua *A. tolteca*	0.619		91.93	91.52	118.17	30.35	129.35	47.27	12.23	14.04
	Managua *A. citrinellus*	0.523	0.310		76.67	66.21	87.39	117.74	52.07	99.75	78.19
	Managua *A. labiatus*	0.101	0.410	0.494		34.42	118.70	130.66	64.58	101.07	86.28
***p*-value**	Masaya *A.* cf. *citrinellus*	0.403	0.424	0.508	0.770		140.41	105.36	84.02	126.46	109.24
	Masaya *A.* cf. *labiatus*	0.593	0.705	0.433	0.313	0.215		108.94	59.78	26.93	33.56
	Nicaragua *A. citrinellus*	0.741	0.870	0.626	0.385	0.523	0.546		162.37	117.30	138.25
	Nicaragua *A. labiatus*	0.445	0.130	0.408	0.518	0.463	0.509	0.218		58.87	35.42
	Xiloá *A. sagittae*	0.601	0.925	0.321	0.379	0.321	0.857	0.768	0.263		24.03
	Xiloá *A. xiloaensis*	0.396	0.934	0.574	0.539	0.479	0.824	0.232	0.791	0.927	
